# The Southern Frontier of the Meroitic State: The View from Jebel Moya

**DOI:** 10.1007/s10437-014-9164-5

**Published:** 2014-09-02

**Authors:** Michael Brass

**Affiliations:** Institute of Archaeology, University College London, 31-34 Gordon Square, London, WC1H 0PY UK

**Keywords:** Cemetery, Correspondence analysis, Jebel Moya, Multi-distance scaling, Pastoralism, Sudan

## Abstract

The site of Jebel Moya, excavated in the early twentieth century, represents arguably the largest pastoral mortuary complex in Africa. Jebel Moya is resituated in relation to the neighbouring Meroitic-era agro-pastoral settlements and the only known Meroitic trading station (Sennar) in the southern Gezira Plain, Sudan. It is the first time that the known localities in the southern Gezira and southern Meroitic cemeteries have been compared, in an attempt to elucidate the different social organisation reflected in mortuary assemblages between the core and the periphery of the Meroitic State. New questions are posed for (1) the applicability of mortuary theory to pastoral cemeteries, and (2) the nature of zones of interaction on the frontier of the Meroitic State, through the application of new statistical and spatial analyses of the mortuary assemblages and the site’s reinterpretation as a pastoral, instead of an agro-pastoral, mortuary complex.

## Résumé

Le site de Jebel Moya, fouillé au début du 20ème siècle, représente vraisemblablement le plus grand complexe mortuaire pastoral d’Afrique. Nous reconsidérons le site de Jebel Moya par rapport aux occupations agro-pastorales de l’ère Méroïtique ainsi qu’à l’unique comptoir commercial Méroïtique connu : Sennar (sud de la plaine de Gezira, Soudan). Pour la première fois, les localités du sud Gezira et les cimetières du sud Méroïtique sont comparés. L’objectif étant de tenter d’élucider les différentes organisations sociales que reflètent les assemblages mortuaires du cœur et de la périphérie de l’état Méroïtique. Cette étude soulève de nouvelles questions (1) sur la pertinence des théories mortuaires dans un contexte de cimetières pastoraux, et (2) sur la nature des zones d’interactions à la frontière de l’état Méroïtique au travers de l’application de nouvelles analyses statistiques et spatiales des assemblages mortuaires d’une part, et d’autre part, de la réinterprétation du site en tant que complexe mortuaire pastoral au lieu d’agro-pastoral.

## Background and Aims

**Fig. 1:**
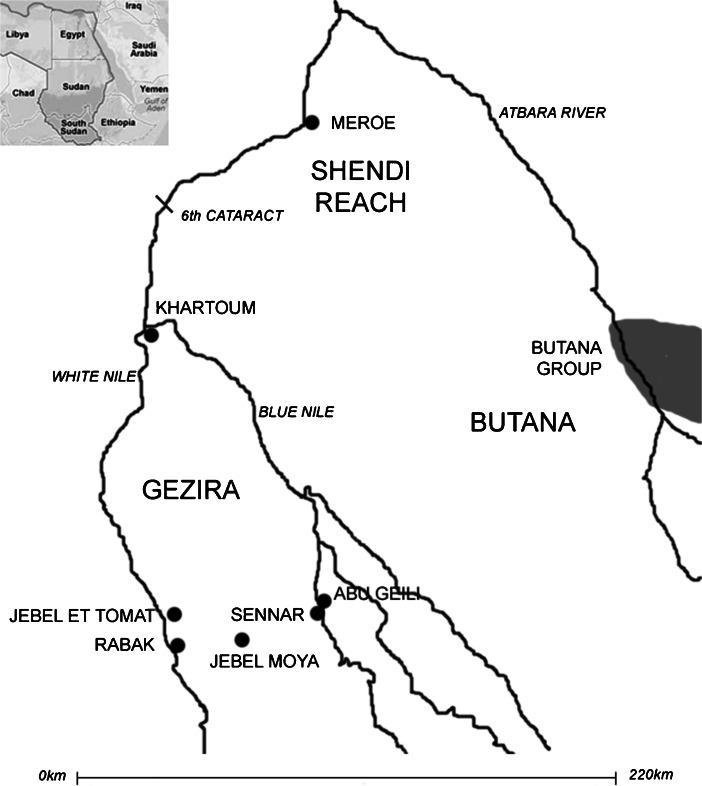
The location of Jebel Moya in the southern Gezira Plain below Khartoum. (Adapted from Edwards 1989, Fig. 1 and Winchell 2013, Fig. 1.2)

**Fig. 2:**
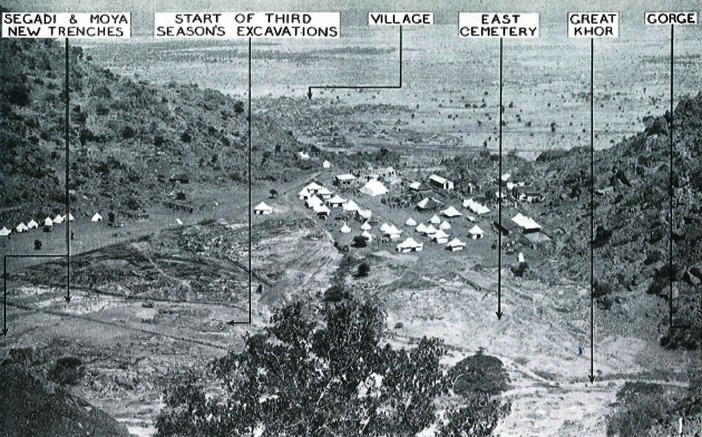
View during the third field season (December 1913) looking north across Site 100. (From Addison 1949, Plate II)

Sir Henry Wellcome, founder of the Wellcome Trust, excavated the valley termed Site 100 in the northeastern portion of the Jebel Moya massif, in the southern Gezira Plain of the Sudan, over four field seasons from 1911 to 1914. The massif is situated between the Blue and White Niles, approximately 250 km south/southeast of the confluence of the Niles at the Sixth Cataract by Khartoum, and some 30 km to the east of the claimed Meroitic trading station and later Medieval Funj kingdom seat of power at Sennar (Fig. 1). At the conclusion of the original four seasons of fieldwork, a fifth of the 10.4 ha had been excavated (Fig. 2) (Addison 1949). No further excavations have been undertaken, apart from a couple of test trenches dug by J. Desmond Clark’s team during a brief visit in the early 1970s (Clark and Stemler 1975).

Overall, 3,135 human burials in 2,791 designated graves were both recorded and excavated, making the site the largest pastoral mortuary complex in sub-Saharan Africa, with the vast majority of the burials dating to the site’s final phase from the first century BC until the mid-first millennium ad (Brass and Schwenniger 2013). The excavated materials and expedition records were shipped to the UK. The surviving anatomical remains are curated at the Duckworth Laboratory (University of Cambridge), the excavation records at the Duckworth Laboratory and at the Griffiths Institute (Oxford), a representative pottery sample at the British Museum and Petrie Museum (London), and the majority of the known surviving artefacts at the Museum of Anthropology and Archaeology (University of Cambridge). The site has been largely ignored due to (1) the unusually large number of workmen employed, which hindered detailed recordings of materials and remains by the field excavators, and (2) the existence of two different chronologies proposed by the author of the archaeological site report, Frank Addison, based upon the same pottery assemblages and stratigraphic distribution of graves (Addison 1949, 1956; Brass 2009; Gerharz 1994). Consequently, limited attention has been paid to the archaeology of the Gezira Plain in favour of the territory covered by the Meroitic State down to the Shendi Reach (Edwards 2007; Salvatori 2012; Fernández *et al.* 2003).

Despite the positional and material nature of Jebel Moya, no attempt previously has been made to elucidate the nature of social organisation as reflected in the mortuary assemblages and how it relates to the changing nature of the socio-political orders and the processes affecting their cultural evolution in the southern Gezira Plain. The nature of the site, and the wealth of inadequately described and interpreted artefacts, provide a unique opportunity to re-evaluate the social archaeology of the presently poorly represented areas south of Khartoum in the southern Gezira Plain. Consequently, the following questions are being addressed in the ongoing programme of research:

- What is the lifespan of the use of Site 100, and how does its use change over time?
- To what extent are phenomena, including burials, burial types, burial assemblages, body orientation and posture, age and gender spatially clustered within the cemetery, and how do they allow for informed social analysis of behaviour?
- Does the distribution of grave goods spatially and temporally demonstrate significant social differentiation in comparison to mortuary assemblages elsewhere in the Sudan and southern Egypt?

Placing Jebel Moya in a secure temporal context, through attribute analysis and optically stimulated luminescence (OSL) dating of the pottery assemblages curated at the British Museum, was a necessary step to allow for informed social analysis of change over time (Table 1) (Brass and Schwenniger 2013). In this paper, the applicability of the wider body of mortuary theory to Jebel Moya is discussed, and preliminary results are outlined from spatial and statistical analyses revealing the existence of seemingly unique spatial neighbourhoods in the northeastern sector of the cemetery. Finally, comparisons are drawn between the social aspects of the vast majority of the burials and burial assemblages from all the sectors at Jebel Moya, dated to Assemblage 3, and contrasted with nearby sites, as well as two Meroitic localities from the Shendi Reach above Khartoum, to facilitate an emerging understanding of the social nature of mobile communities on the southern periphery of the Meroitic State (300 bc–ad 350) for the first time.

**Table 1:** A chronological framework for Jebel Moya as determined by OSL dating of pottery from the British Museum

Phase	Characterisation	Date
Assemblage I	Small-scale, periodic occupation	Sixth or early fifth millennium bc
Assemblage II	Small-scale occupations over an extended period, coincident with Middle and Classic Kerma periods to the north	Mid-second to mid-first millennium bc
Assemblage III	Vast majority of the burials, spanning the Middle to Post-Meroitic phases of the Meroitic State	First century bc to mid-first millennium bc

(Summarised from Brass and Schwenniger 2013)

## Complexity Theory and Mortuary Assemblages

Burial grounds are physical and symbolic clusters of elements with internal and external sets of boundaries. As originally acknowledged by Morton Fried (1967, p. 112), although burial practices may reflect aspects of socially stratified societies, differential status may not be readily apparent in the resultant material traces. Aside from attritional instances such as casualties sustained in warfare, different members of society are disposed of according to social norms. Key factors such as increasing population density or dispersal and proportional differences in age and sex do not necessarily correlate with ratios of burial types in a cemetery. An additional challenge lies in determining the changing interrelationships between kinship groups, where shifting alliances or increases/decreases in power may provide spatial or material pointers that assist in deciphering and reconstructing mortuary data. Social power is situational, fluid, overlapping and intersecting (Chapman 2003; Mann 1986; Pauketat 2007) and is reflected in the material assemblages, but one component of the mortuary rites displaying and expressing degrees of affiliation to different groups in the community (social advertising). These expressions can result in material differences between spatial clusters of burials (Wobst 1977). Mortuary practices may therefore also involve considerations of territoriality or spatial clustering within cemeteries which may indicate elements of relatedness or desired affinity (Di Lernia and Manzi 2002; Dunham 1999; Smith *et al.* 2002).

While the mortuary rites of some early societies may contain material expressions of inequality, Paynter (1989) also cautions that this is not indicative of inheritable elite roles and therefore formal vertical stratification. Power can be heterarchical, “counterpoised rather than ranked” (Crumley 1995, p. 3), with fluid heterarchical relationships permissible at given scales within broader hierarchical social systems. The heterarchical interrelationships can be seen as a mechanism through which different social units aggregate (McIntosh 1993). At the core of the interdependent relations which make up social practices are issues of dominance, influence and power. How these principles are structured delineates and shapes the behaviour of individuals, groups and communities. Although certain behaviours are legitimated by the actions of one or more dominant sectors of the society, potentially creating a mirage of independence, there are counter-trends and push-backs by lesser dominant peoples or subversives which also form part of the interwoven social fabric (Reid and Lane 2004).

There is therefore no direct, inherent correlation between complexity as a conceptual tool and the expression of formalised inequality, which goes against the implicit grain of previous neo-evolutionary studies that not only tended to look for patterns in differences and similarities between societies, but also regarded the monopolisation of power and resources as being reflected in the variation of grave goods (McGuire 1983; McGuire and Paynter 1991; Paynter 1989). It is through the manner of burial—the actions of and the social make-up of the mourners—that the deceased is represented and identified (Parker Pearson 1999; Stevenson 2009), a point which had been previously iterated by Brown (1981) but which was not fully taken up within the processual investigations utilising the Saxe–Binford approach.

Funeral rites sustain, negotiate and revitalise the social order and identities (Bloch and Perry 1982). In addition, some societies in which status is *achieved* view it as socially acceptable to deposit high-value goods in the graves with older individuals (Binford 1972, p. 226), while young adults (even those who have already achieved some form of standing) may not be buried with similar goods due to it being regarded as “culturally unacceptable to translate grief into grave-good abundance” (MacDonald 2001, p. 708), a potential component of embodied experiences of grief (Tarlow 1999). Moreover, some grave assemblages might be attributable to inheritance (Chapman and Randsborg 1981, p. 13), which may explain some of the poor quantity of grave goods in prehistoric semi-sedentary pastoralist societies in the eastern Sahara (*e.g.*, Kobusiewicz *et al.* 2010).

In an attempt to circumvent these issues, Stephen Savage (1997) developed a diachronic model based on intergroup competition. He applied Ortner’s (1984) Action Theory in hypothesising that short-lived chiefly and sub-chiefly lineages were in competition with each other, with different lineages predominating at different times. He suggests that these actions are reflected in the spatial clusters and material burial goods found at the fourth-millennium bc cemetery N7000 at Naga-ed-Der in Upper Egypt (Savage 1997, p. 228). Drawing upon ethnography of the Central African Mango, who are said to have divided their burial spaces along similar lines (Maes 1924), Savage equates Ortner’s successive “acting units” or “social types” with Predynastic social structures based on kinship. Their fluctuating power was attributed to the expression and/or suppression of organised activities designed to enhance social status. *Longue durée* change is therefore a side effect of continual action.

Such fundamental beliefs and social actions are expressed and partly materialised during the ritual communications and actions comprising the mortuary rites. The behavioural signals in question take both verbal and non-verbal forms. They transform the environment through their repetition and stylised actions, which are effectively rule-bounded performances merging the past with the present (Robb 2007). The rituals, in which individuals are assigned particular roles dependent upon their particular status, serve to both demystify and mystify the meanings behind why an action is taken and/or why an event occurred; the meanings themselves are context-dependent. As such, the rate of change is quicker for social actions other than mortuary rituals (Bloch 1989).

Even so, the symbolism behind the mortuary rituals changes over time and an explanatory framework is needed on how to recognise and interpret. Categories of symbolism within a material context include the goods deposited with the body or bodies (in cases of multiple burials in a grave), treatment of the corpse, where the grave was situated and how it was marked, and the placement in the landscape of the cemetery. Social advertisement is achieved not only at individual or group level within the cemetery but also at community level: A cemetery or cemeteries can be situated along trade routes (Acacus Mountains, Libya, Di Lernia and Manzi 2002), at a particular sacred locale (*e.g.*, Giza in Egypt, Roth 1993), or associated with a naturally prominent feature in the landscape (*e.g.*, Site 100 in the Jebel Moya massif).

## Spatial and Statistical Analyses of the Jebel Moya Mortuary Complex

Of the 3,135 human burials, 1,108 (35.3 %) have associated recorded items. The original excavation records were re-examined and their information captured in a new Register of Graves, and cross-correlated with both Addison’s original Register and the results of a re-sexing of the extant skeletons curated at the Duckworth Laboratory, undertaken by the curatorial staff. For Jebel Moya, the new Register of Graves contains the following information: excavation season, burial number, square, distance below the ground surface at the time of excavation, distance above/below Addison’s datum point, grave type, burial orientation, length of body, body posture, degree of depositional disturbance, field sexing, laboratory sexing, present-day location of the artefacts and the nature of any accompanying burial goods.

The resulting information and the original grave distribution map were linked together in an ArcGIS database, enabling the plotting of multiple variables such as the quantity and spatial variability of grave goods, the number of adults and their sex, the number of infants, artefacts of social significance and artefacts made from a wide variety of raw materials from different sources. It assists in identifying structuring mortuary principles—how material culture was combined and articulated within graves—and therefore how pottery and other burial goods were used in certain contexts. For the purposes of this paper, these include the comparative relative density of grave wealth in different sectors of the cemetery, the types of artefacts accompanying male and female burials, cluster analyses and cross pair correlation function to look for potential spatial neighbourhoods.

A null hypothesis was devised to determine the likelihood of the cemetery’s basic layout having been established at the outset, or whether it grew organically using pinpoint analysis. If it grew organically, it was expected that the composition of artefacts in different areas would differ in frequency and make-up. There were very few differences in the occurrences of artefact categories across and within the examined sectors, with the possible exception of more bracelets in the east and north-east and more lipstuds and armlets in the southwest. Nor were there any differences in the sub-surface construction of the graves apart from some being rectangular and others oval with no valid differential spatial distribution pattern. There is no conclusive evidence for differential homogeneity between any areas of the mortuary complex.

### Relative Risk

The density of burials with goods to burials without goods was calculated across the excavated areas using relative risk (RR) in the program R (Bevan 2012). R is an integrated suite of software facilities for data manipulation, calculation and graphical display which permits a wide variety of statistical and graphical analyses to be undertaken such as clustering, classic statistical tests, distance scaling and matrix calculations. What is being measured through the application of RR for the first time in Sudanese archaeology is the ratio of cases to controls; the kernel density is used to map this smoothly across the study region. The relative risk’s cases are the burials with goods, and the controls are the burials without.

**Fig. 3:**
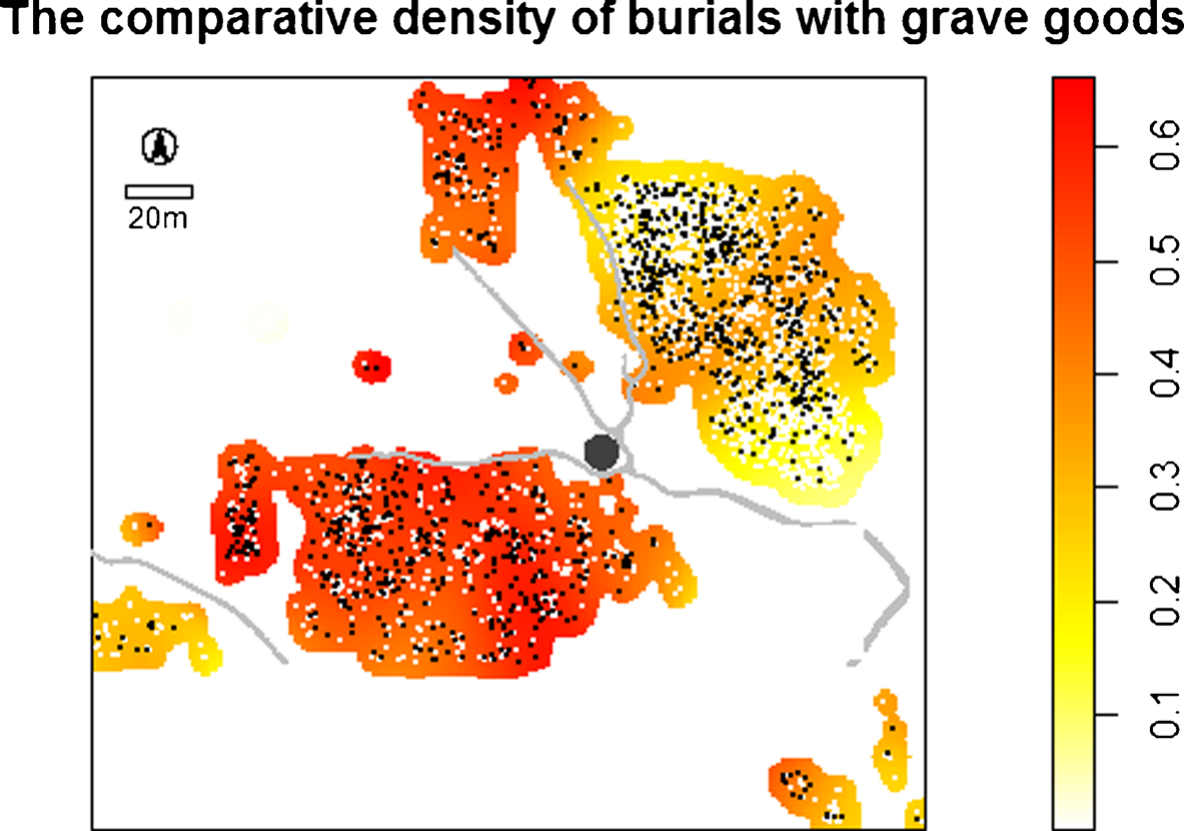
The relative density of burials with goods to burials without goods across the cemetery. The *black dots* represent the burials; the *grey lines* are eroded water courses, and the *black dot* in the middle is a prominent boulder. The density is greater in the southwest and north (>0.5) than in the other sectors (published in full colour online)

In the southwest, the red zones on the plot (Fig. 3), the ratio of burials with goods to burials without goods is much higher (>0.5) than in the northeast sector. Therefore, while the northeast sector is richer in certain kinds of finds made from non-local raw materials, fewer burials have grave goods here, *i.e.*, the wealth distribution seems to be more skewed, which may represent different rules of social engagement in mortuary practices.

### Inverse Distance Values

Speculation about imported items being present contains inherent assumptions on the nature of trade relations in the southern Gezira and potentially disguises the form that trade interactions may have taken. It is impossible to determine with any degree of certainty that items, apart from the very few scarabs and a couple of amulets, arrived in the area in their final form. Instead, the approach taken here is that only potential sources of origin for the raw materials from which the items were manufactured can be reasonably deduced and acted upon for analysis. Inverse distance value (IDV) is therefore defined here as the weight (value) assigned to a material which diminishes as the distance from the area/region of origin decreases.

Artefacts were manufactured from alabaster, bone, brass, bronze, carnelian, chalcedony, chert, copper, cowrie shells, crystal, diorite, faience, feldspar, flint, glass, gold, granite, iron, ivory, jasper, limestone, limonite, marble, natrolite, pottery, quartz, sandstone, shells, silver and steatite. Values of 1–4 were assigned based upon degree of accessibility to, and distance from, potential raw material sources, where 1 is local within a 20 km radius, 2 is the banks of the Niles, 3 is central and northern Gezira and 4 is from Meroitic territory or through Meroitic trade networks.

The overall IDV for each burial was calculated on the presence and numbers of items made from the different raw materials. The IDV for each sector of the cemetery was then determined (Table 2). While items made from imported raw materials appear only in the east and northeast, with the exception of marble lipstuds and one occurrence of faience beads in the southwest, the values of the southwest and northwest sectors also indicate that there are different concentrations and materially (possibly ideologically) represented manifestations of wealth, across the cemetery.

**Table 2:** Breakdown of the total number of human burials, and human burials with grave goods, in the different sectors of the site

Sector	Total burials	Burials with goods	Inverse distance value
South	49	17	41 (0.87; 0)
Southwest	804	427	1,758 (2.16; 1)
West	17	4	6 (0.35; 0)
East	852	217	1,080 (1.26; 0)
Northwest	171	85	356 (2.09; 0)
Northeast	1,185	336	2,137 (1.8; 0)
Non-assigned	57	22	86 (1.48; 0)
Total	3,135	1,108	

IDV is the total for each sector with the accompanying mean and median values per human burial in brackets

### Spatial Neighbourhoods

Correspondence analysis, non-metric distance scaling and distance-based metric multidimensional scaling techniques were run, but no valid clusters of burials based upon their accompanying artefact categories were detected.

Pair correlation function, which examines how the density of objects vary as a function of distance, was subsequently used for point pattern analysis in R to determine the spatial configuration for richer burials and less rich burials, where each point represents a burial in two-dimensional space. Richer burials are defined as having an IDV of 10 or more. Two tests were run against the burials of each sector of the cemetery: (1) The spacing of the richer burials *versus* each other, and (2) the spacing of richer burials against less rich burials, with a randomisation of rich and non-rich burials forming a delineated set of values against which the burial data were calculated.

**Fig. 4:**
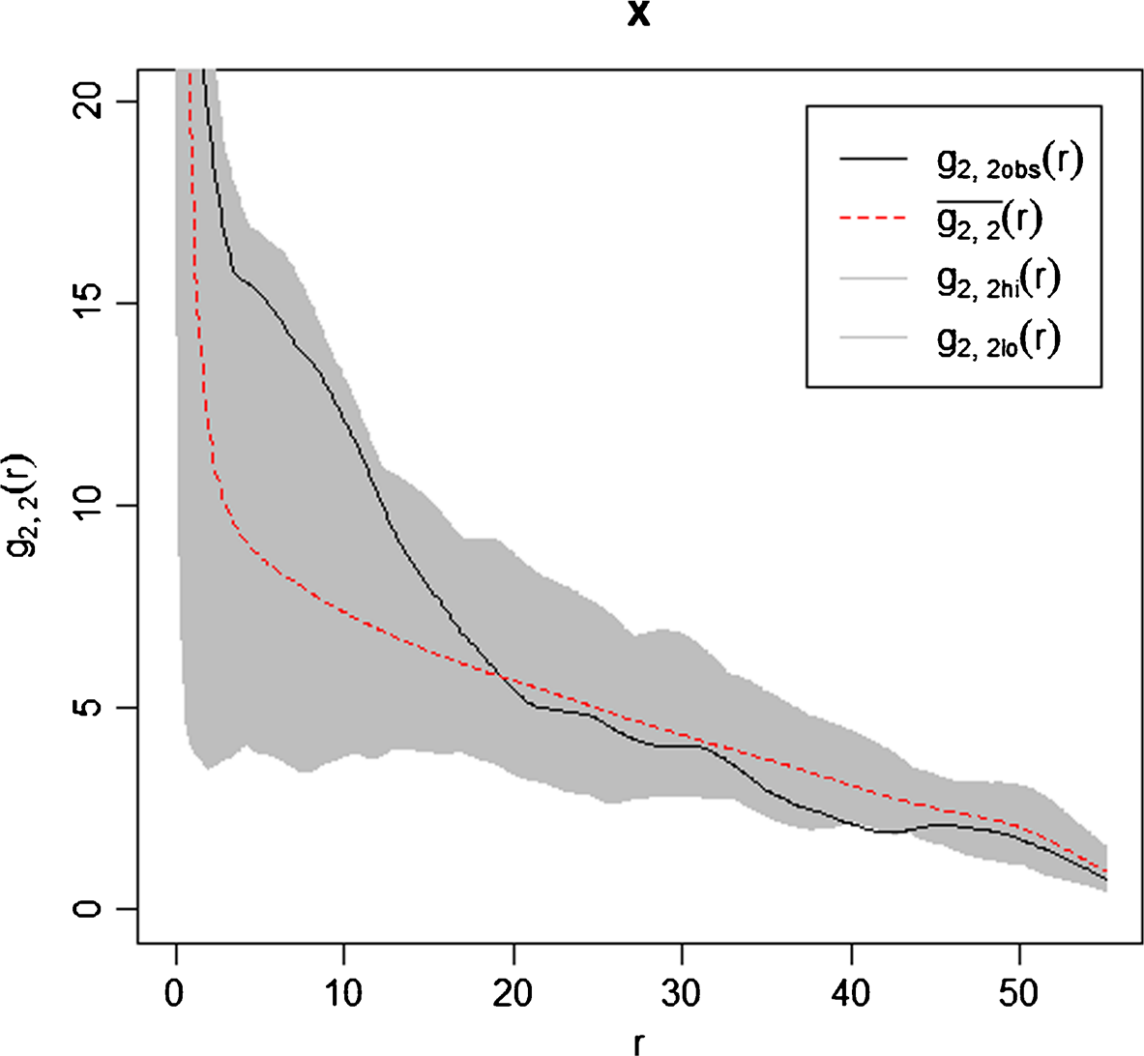
Pair correlation function (PCF) plot of richer burials *versus* each other in the southwest sector of the cemetery. *X*-axis: metres out from any given rich burial. *Y*-axis: an estimate of the density of neighbouring rich graves for difference distances along the *x*-axis. *Red dotted line*: average PCF value for 99 random sets. *Black line*: the burial data. *Grey shaded area*: wider envelope of possible values from the random sets

**Fig. 5:**
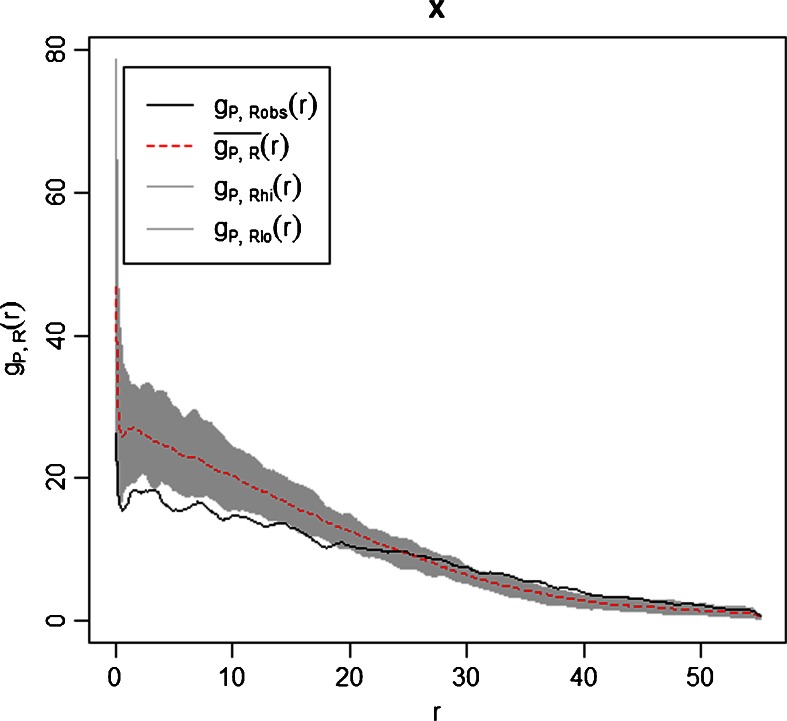
Pair correlation function (PCF) plot of richer burials *versus* less rich burials in the northeast sector of the cemetery. *X*-axis: metres out from any given rich burial. *Y*-axis: an estimate of the density of neighbouring rich graves for difference distances along the *x*-axis. *Red dotted line*: average PCF value for 99 random sets. *Black line*: the burial data. *Grey shaded area*: wider envelope of possible values from the random sets

For the first test (Fig. 4), the spatial distribution of the richer burials shows no significant patterning, with no suggestion of standardised distances separating the burials. However, an interesting spatial neighbourhood phenomenon in terms of the distribution of richer *versus* poorer burials was observed uniquely for the northeast sector in the second test (Fig. 5): There is some inhibition between richer and poorer burials at distances of up to 20 m, where the line representing the data values dips below the randomisation envelope; the former distance is the result of determining that there is poorer burial density out from each richer burial to a radius of 20 m. At larger distances, there is significant clustering where the data values are greater than the randomisation envelope. Therefore, there are fewer poorer burials than expected at short distances from richer burials, but a greater number of poorer burials than expected beyond; in other words, there is a halo of poorer burials at a distance of 20–40 m from richer burials.

These results raise interesting questions on the meaning of spatial relationships. They suggest a degree to which it was not deemed appropriate to place poorer burials very close to the members of the communities buried with richer accompaniments in the northeast sector, *i.e.*, there is clearly some kind of spatial neighbourhood. One possibility is that there may have been some form of non-permanent marker for the rich graves, indicating to the community where not to bury while the marker lasted and until the position of the grave(s) faded from memory. Such a spatial arrangement is unknown in the African pastoral ethnographic literature where the structures of the burials are all simple oval or rectangular graves with no distinguishing features other than their burial goods. Although a previously unknown phenomenon is postulated, these burials cannot be assigned uncritically to hereditary elites or to a dominant clan lineage without critical analysis of how different aspects of social organisation are represented (McHugh 1999), especially as there is a lack of spatial clusters of both burials and accompanying grave goods. Heterarchical and hierarchical ideologies appear to be intertwined with the unique aggregation of social units in the cemetery. Just how these rule-bound performances and practices were constituted and what the wider implications are for informed social analyses of change in the mortuary record of Jebel Moya remain under investigation.

## Jebel Moya as a Pastoral Mortuary Complex

Rachel MacDonald (1999) compared samples of teeth from ethnographically and archaeologically known hunter–gatherer (Efe, Du Chaillu, Gwisho, Chencherere, Shum Laka), pastoral (Somali, Adrar Bous, Jebel Moya) and agricultural populations (Haya, Teita, Igbo, Tellem). She partly filled a gap in Mukherjee *et al.*’s (1955) report which did not cover the dental and post-cranial remains, with the consequence that data were not compiled on population demography, health and diet.

**Fig. 6:**
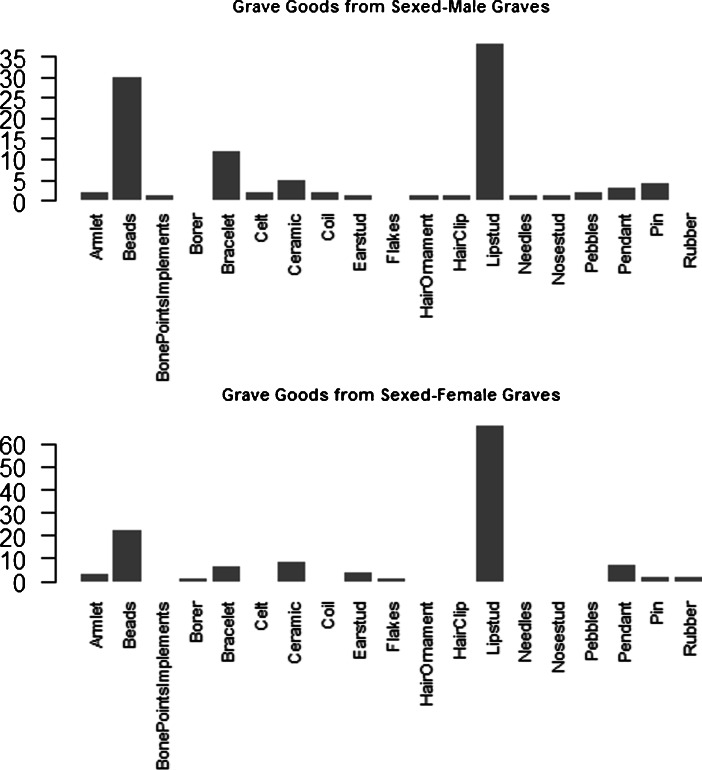
Frequency of burial objects from Duckworth Laboratory sexed female and male burials

The Jebel Moya dental samples, totalling 2,411 teeth, were encased in a fine layer of cemented sand. High levels of enamel chipping were only observed in the Jebel Moya sample. Enamel damage was altogether more severe compared with the other population samples, with the molars and premolar cusps chipped and/or broken. The damage could sometimes be observed on several teeth per individual, and it was particularly common on the lingual cusps. MacDonald (1999, p. 118) suggested the damage was due to diet as “the use of teeth as tools would tend to favour the damage of enamel on the buccal and labial surfaces of the teeth.” She ruled out it being a consequence of wearing lipstuds on the basis of Addison’s assertion that they were worn by females. However, the asexual nature of the distribution of burial goods with skeletal remains subsequently resexed by the Duckworth Laboratory (Fig. 6) suggests it remains a valid possibility as a contributing factor.

There are also wear patterns on the labial surface of some of the anterior dentition as well as wear-facets on lipstuds. Dental manipulation was common and included the deliberate removal of some teeth. Although tooth removal is not always the result of dental manipulation, around 12 % of individuals with labret wear-facets still possessed their anterior teeth. The lipstuds were most commonly worn through the bottom lip and were not always deposited in the burial assemblage: Of MacDonald’s determined wearers, 29 % had no surviving accompanying lipstuds in their grave, which may possibly be due to either removal prior to burial (during mortuary practices or towards the end of the person’s life) or being buried with now decayed wooden lipstuds.

Dental caries occur when the pH of the oral environment remains low, below 5.5, for a prolonged period of time, causing the dental enamel to become demineralised. There is a linkage between dietary sugars and caries, and the incidence of caries has increased since the advent of agriculture. The incidence of caries in the Jebel Moya sample, expressed as a percentage of the total number of teeth examined, is 0.2 % which groups together with known pastoral societies from Adrar Bous, Riet River and Kakamas (MacDonald 1999, p. 161). By contrast, the recorded incidence amongst a sample of 581 teeth from more agriculturally inclined Meroitic Nubia was 15.1 %. Furthermore, the Jebel Moya caries occur most frequently on the third molar in line with other pre-agricultural communities, whereas caries occur most frequently on the second molar in predominantly agricultural societies.

Abscesses in pastoralist populations are due to dental attrition (mastication) or the wearing of lipstuds/labrets. Calculus formed on 10.6 % of pastoralist teeth and 37.1 % of agriculturalist teeth in MacDonald’s sample groups. Pastoralists had the least occurrence of antemortem tooth loss with 32.6 % of the total number studied (MacDonald 1999, p. 174). For Jebel Moya, the ritual removal of anterior teeth can be taken to account for much of the instances of the resulting good dental health.

Further reinforcing the biological evidence for pastoral populations at Jebel Moya is the lack of archaeological evidence for artefacts associated with harvesting, such as sickles and hoes. The faunal remains consist of cattle, dog and goat, of which cattle and dog were the only animals in their own graves, twinned with a human burial or, in the case of cattle, had parts of the animal included with a human burial. Several clay cattle figurines were also found in non-burial contexts.

## The Nature of Meroe’s Southern Frontier

Attempts have been made to place the Meroitic State in a Sahelian context in recent years. They draw upon the Southall’s ethnographic model of segmentary lineage societies (Southall 1988a, b, 1999) and upon studies of the sultanates of the Funj (based at Sennar along the Blue Nile from the sixteenth to early nineteenth centuries ad) and Keira (Darfur, seventeenth century ad) (Edwards 1996, 1998b; Fuller 2003). In such segmentary systems, ritual and political influence have contrasting spheres of control: Ritual activities in the peripheral areas are in constant flux, while the seat of political authority is centred on the core domains of the territory held in place by checks and balances of ritual sanction and institutionalised interdependence.

It is in the Edwards and Fuller hypothesis, termed the Sudanic Model (Edwards 1998b), that Meroitic political authority has been characterised as: (1) dependent on establishing and maintaining long-distance exchange and (2) appropriating and assimilating Egyptian and regional religious rites. The importation and redistribution of high-valued items and slaves from the south to the north and into Ancient Egypt established the ritual hegemony of the Meroitic royalty based at Meroe, and integrated distinctive geographical and political areas to the north and south (Edwards 2007). Formal political ties were further cemented by titles and court privileges given to regional elites and by intermarriages with female royalty. In return, tribute flowed along exchange corridors to the centre, Shendi Reach, and the capital Meroe.

The application of the Sahelian segmentary state concept to the Meroitic period has resulted in the recognition of the fluid, contextual nature of exploitation and power relations between and within the diverse regions and the social structures. *Contra* Adams (1977), Lower Nubia did not support a large continuous population, and the economic basis was subsistence farming with little scope for long-term surplus (Fuller 2003). Instead, more plentiful arable land was available and cultivated in the Western Butana, which may have been the only area under the personal control of the Meroe royalty (Edwards 1996, p. 90). The royal palaces and the non-food producing centres of the Meroitic core in the Shendi Reach needed to be supported. The complexity, nature and scope of the exploitation have yet to be adequately fleshed out; however, the available data show a veritable mix of agro-pastoral and pastoralist communities living in the Butana at this time (Ahmed 1984; Bradley 1992).

By contrast, little is known about the nature and extent of Meroe’s political, ideological and socio-economic reach southwards into the heart of the Gezira Plain, and beyond. It has been claimed that the gold resources present along the Blue Nile were exploited (Edwards 1998b, p. 191), though the type and location(s) of the exploitation remain undetermined, and slaves were probably brought up through the Gezira from the south. Whether there was the same degree of agricultural exploitation as in the Western Butana, evidenced amongst other things by water construction works, is unknown due to the lack of modern, systematic archaeological surveys in the region. Most of the known sites were discovered by Wellcome’s expedition in the early half of the twentieth century, subsequently supplemented in the early 1970s by brief and somewhat haphazard surveys, particularly in the southern Gezira.

What is evident to date is that the Gezira is distinguished from the areas to the north of Shendi Reach by the comparative lack of status objects produced by the production centres in and around Meroe. This could be explained in terms of cultural or political distinctiveness. However, the status of rural inhabitants must also be considered and how their ability to obtain such objects was impacted by their degree and type of access to exchange networks (Edwards 1996), and how this was represented in mortuary organisation through burial separation between select, potentially highly regarded individuals and their communities. Alternatively, the lack of status objects may have been because such items were not used in similar ways in mortuary rites which, coupled with the lack of adequate surveys and excavations of identified sites, would produce a distorted picture of the nature and subsequent use of the trade relationship between the peripheral local inhabitants and the Meroitic centre.

Meroitic items have been found as far south as Kosti, which is a little further south than Jebel Moya and is on the banks of the White Nile (Eisa 1999). Meroitic objects have also been discovered further near Grisly village, at El Getina, Dinder, Wad Sheneina and at El Tersab, 24 km south of El Getina on a plateau containing Meroitic- and Christian-period burials that have not been adequately excavated (Ahmed and Ahmed 2004; Eisa 1999; Fernández *et al*. 2003). Other small sites have been identified, but it is unclear how many can be confidently assigned to any of the Meroitic periods, especially since the dating rests on questionable ceramic typological methods (Brass and Schwenniger 2013).

The site of Abu Geili is almost 2 km north of the ancient Sennar cemetery (Fig. 1). A village and a cemetery were excavated by Crawford, a member of Wellcome’s expedition, in 1914. The cemetery was dated to the Funj Sultanate. Yet, while the village’s pottery coincides in part with cemetery usage at Sennar, no Meroitic elite items are found in the village similar to those in the Sennar graves (Crawford and Addison 1951). They are probably from two different communities living in close proximity to each other. Crawford dates the village from 200 bc–ad 600, spanning the Classic, Late and Post-Meroitic periods, with its end probably coinciding with the introduction of Christianity in the region (Crawford and Addison 1951, p. 11).

The Abu Geili village also has occupational debris such as potsherds and beads (Addison 1950). Numerous pits were either dug by the inhabitants beneath their houses, or their houses were built over earlier in-filled pits (Crawford and Addison 1951). Overall, there is some wheel-made pottery, but most examples are handmade and were said by Addison (Crawford and Addison 1951, p. 42) to be distinct from the Jebel Moya assemblages, although there were some Jebel Moya wares present (Brass and Schwenniger 2013, Fig. 9). These Jebel Moya wares are from Assemblage 3 and consist of black polished, incised, cord-wrapped stamped and comb-stamped wares.

The cemetery at Sennar was discovered in 1921 on the east bank of the Blue Nile. The remains include carnelian, Lydian stone, faience, glass and quartzite beads, faience figurines (*Bes* and *Amun* ram with the sun disc), pottery and bronze vessels (Addison 1950). The wheel-made red-ware was probably imported from Meroe via the exchange networks, as were the jars of common Meroitic form. The handmade pots are regarded as local forms (Addison 1950). Some forms of the bronze vessels have been found at Meroe West Cemetery. Similar carinated bronze bowls occurred in elite burials at Meroe (Addison 1950), reinforcing the elite exchange network hypothesis of Edwards (1996).

A circular grave was subsequently found in 1925 on the west bank. All but one of the 40 pots were black-ware with no external decoration, unlike the Jebel Moya black-ware (Addison 1950). Addison claims there are affinities with the Abu Geili pottery where Jebel Moya pottery types have been found, making the sites at least partly contemporary. The grave does not contain any distinctly Meroitic pottery, but one should not draw firm conclusions based on one grave. Three more graves were found nearby by Arkell—one Funj, one post-Meroitic and the other Meroitic—suggesting the former presence of a small cemetery in the vicinity which was damaged or destroyed by riverine action (Addison 1950). The post-Meroitic grave contains black-ware with incised decoration externally and internally, which contrasts with the black-ware from Jebel Moya which is incised externally.

The associated Sennar settlement remains undiscovered. The materials found in the cemetery differ from those found at the nearby habitation site of Abu Geili (Addison 1950). Addison proposed that the settlement held a similar status to that of Faras in Lower Nubia; however, the extent of the cemetery has yet to be determined, and the precise number of imported items is unknown, since many objects were lost over the course of time, and others were onboard a ship that sank en route to England. Addison further hypothesises that Sennar was abandoned during the late second century ad, his reasoning being that this is when the perimeter of the Meroitic Empire was beginning to break up and decrease in geographical size. This dating can be questioned on the basis of new archaeological knowledge which dates the break-up of the Meroitic State to the late third and early fourth centuries ad, and on the basis of the Jebel Moya dates on Assemblage 3 pottery.

Crawford and Addison (1951) claim that a sherd from an enclosure at Sagadi, 12 miles (ca. 19 km) northwest of Jebel Moya, resembles sherds from Jebel Moya’s Assemblage 3. Other finds include stone rings, armlets and maceheads which Crawford claims are similar to their counterparts at Jebel Moya. These remains have not been re-examined since, to verify their validity.

What seems to be clear, from the existing evidence, is that the southern boundary of the Meroitic State was in the region of Sennar on the Blue Nile, with the incorporation of the Gezira into the network of power relations (Adams 1977, pp. 341–342). Further information on the nature of social organisation at the Gezira periphery and its differences with the cultural groups comprising the Meroitic Empire can be elucidated, by briefly comparing the mortuary behaviour exhibited at Jebel Moya with the non-elite Meroitic cemetery at Gabati and the form of agro-pastoral burials at Jebel Sabaloka, both in the Shendi Reach, the heart of the Meroitic State.

At the Gabati cemetery, south of Meroe, which dates from the first century bc to the early second century ad, 63 of 74 identified Meroitic graves were excavated with a total of 124 burials (Edwards 1998a). The average density per sq. m was two graves, which is a significant contrast to Jebel Moya where the density reached a maximum of 10 per sq. m with 205 graves in square J.9, K.10 in the northeast. Of the 63, only four retain traces of a black, chipped sandstone superstructure encased by mudbrick. Edwards speculated that the other graves may have been marked by a low sand mound. No such superstructures have been identified at Jebel Moya, where the pastoralists’ graves were oval or rectangular without burial shafts. Two of the superstructures at Gabati have traces of a probable chapel on the east side. The four superstructure burials contained chambers accessed by ramps. By contrast with the variable orientation of the Jebel Moya graves, the bodies at Gabati were more uniformly orientated east–west (in superstructures) or north–south (for the remainder), potentially implying a more uniform local ideology. Furthermore, a larger proportion of human burials contained pottery—62.9 % (Gabati) to 2.41 % (Jebel Moya).

Upstream from Gabati is Jebel Sabaloka, located in the Sabaloka Inlier which is part of the Sixth Nile Cataract, around 80 km downstream from the confluence of the Blue and White Niles. Many of the known Meroitic period remains are located to the southwest of the mountain slopes. The remains consist of simple stone structures and camp sites attributed by the excavators to relatively mobile agro-pastoralists (Suková and Cílek 2012). There and elsewhere are clusters of Meroitic and Post-Meroitic tumuli, some up to 9 m in diameter. Burials marked by cairns also occur on terraces in nearby wadis which also contained settlements. No tumuli are present at Jebel Moya, where the graves differ in design. The Inlier system of wadis, freshwater features, periodic swamps, constricted riverine landscape and hills made for a dynamic zone of interaction between peoples from the heart of the Meroitic State and agro-pastoralists exploiting both its ecological resources and security, and the outlier desert environment.

## Discussion and Conclusion

The establishment of Jebel Moya as a pastoral mortuary complex feeds into the debate on the importance of pastoralism in the Meroitic south. Some earlier scholars such as William Adams took issue with pastoralism being “a major factor contributing to the cultural differences…between Lower Nubia and the southern provinces,” instead viewing herding activities as an adjunct to farming (Adams 1976, pp. 123–24, 161). More recent research has established that a different manifestation of pastoral and agro-pastoral intermediaries integral to exchanges between a North African state and societies further afield is subsequently to be found in the Butana during the time of the Meroitic State (200 bc–ad 400). The western Butana is suitable for agricultural exploitation while the eastern and southern Butana have better grazing resources, based on the different distribution of soil types (clay plains *versus* the western Butana’s sandstone semi-desert) and differential settlement patterns (Ahmed 1984, pp. 79, 279).

The agriculture of the western Butana formed the subsistence underpinning of the Meroitic state centred on the alluvium soils and the wadis, possibly through seasonal patterns of exploitation (Ahmed 1984, p. 103). Interactions between what has been defined as nomadic populations (Al-Hakim 1972, p. 645; Edwards 1989, p. 149) and sedentary agriculturalists occurred in the western and northern Butana. This most probably occurred as the nomadic populations migrated northwards, during the onset of the monsoon season to escape the tsetse fly belt, and utilised the available grazing at the edges of the wadis from the areas along the river margins of the Blue Nile (below the Sixth Cataract) and the Shendi Reach (above the Sixth Cataract), and in the northern Butana, (Bradley 1986, p. 233–36). Beyond these generalised models, the actual archaeological scale and changing nature of interaction between different forms of agriculturalists, agro-pastoralists and pastoralists within a State context has been little studied for the Butana. However, a generalised understanding of the ecological conditions of the different Nubian regions provides a framework within which certain high-level questions can be posed regarding population movements and settlements.

The southern Gezira Plain comprises dense tree, bush and grass growth with predominantly perennial grasses. The modern annual rainfall around Sennar, to the east of Jebel Moya, is *ca.* 400 mm. The landscape changes gradually to the north as the rainfall decreases, from clay thorn and grasslands to semi-desert grasslands. The modern placement of Jebel Moya is near this transitional belt and is believed to have been the same during the Meroitic periods.

The geological formation of Jebel Moya itself is an outcrop of the Basement Complex piercing through the Sandstone Formation and its overlay. The groundwater aquifers are a consequence of the outcropping of the Sandstone Formation and Basement Complex near Khartoum, acting as a barrier to the underground flow of water originating from the Blue and White Niles. The aquifers reside in the Sandstone, and access to them requires digging through more than 10 m of clays, gravels and sands. These aquifers come near and to the surface at few places in the form of sweeps and springs where there were outcrops of the Basement Complex in the southern Gezira, which created attractive conditions for settlement in the nearby vicinity, particularly during the dry summer (Williams and Adamson 1982, p. 135). Environmental samples are unfortunately lacking from Site 100. Therefore, the new radiometric dates assist in providing a chronological framework through which the archaeology can be cross-correlated with the broader geological trends for the Gezira Plain.

Such localities would have included the pastoral mortuary complex of Jebel Moya and the agro-pastoral locality of Jebel et Tomat to the northwest (Fig. 1), situated between two hills. These jebels stood out in a flatish landscape where such hills were few and far between, and where there was also ready access to water for cattle and clay for pottery. For mobile peoples, access to water and pasture are critical, and their security is key to the establishment and maintenance of social relationships both within and beyond the local community by means of material exchanges (Hodgson 2000).

Edwards (1996, p. 91) hypothesised that the cemetery of Jebel Moya may have been the result of communities being forced into the mountain range by raiding conducted by the Meroitic State, or its local elites, in this frontier zone. However, such a model would now require the raiding to continue over the course of up to four centuries and across the time of the breakup of the State. Nor would it account for the number of burials from what must have been a fairly large mobile population or populations over a sustained period of time. As such, this hypothesis is no longer viable. It is more plausible that there were seasonal pastoral movements occurring in the Gezira Plain coinciding with the seasonal shifting of the monsoon belt. Furthermore, the inhabitants of the southern Gezira were most likely a critical part of a zone of interaction, between settled agro-pastoralists and likely Meroitic trading stations along the Niles and pastoralists on seasonal migrations from the south, coming up to hook into existing trade networks across the southern Gezira frontier.

The paucity of excavated sites and extensive, detailed survey works in the Gezira make the study of Meroitic exchange systems difficult. As a result, little is known about the outbound flow of prestige goods making their way down to the local populace whose remains have been found so far. There is a distinct lack of elite burials found to date. By contrast, the inward flow of tribute items to the Meroitic centre from where they were distributed onwards is better known: gold, ivory, ostrich feathers, skins and slaves (Edwards 1996, p. 90). Although the extent, nature and localities of the gold extraction are as yet undetermined, this does not have to necessitate direct exploitation; it does necessitate access to the resources which can be through intermediaries. If there was a large trading outpost at Sennar, then the area to the west, including Jebel Moya, could have served as a crossroads, and the mobile populations living in the southern Gezira could have acted as the intermediaries/conduits. The sites of Jebel Moya, Sennar and Abu Geili (the latter two on the banks of the Blue Nile) are the largest known sites dating to the Late Meroitic and Post-Meroitic periods.

Jebel Moya’s prominence standing above the flat plain and its geographical location place it in the frontier zone of the little known, southwestern border region of one of the earliest states south of the Sahara belt. There appears to be a context of pastoral societies using Jebel Moya as their cemetery and of long-standing trade exchange centering on the southern Gezira between the Meroitic State and the local communities and/or traders. It also appears to have led to one of the only known examples of a large pastoral cemetery at the interface with a State in Africa.

While the southern Gezira is not the only known zone of interaction at an interface between the territorial boundaries of the Meroitic State and savannah or desert communities, Jebel Moya is the largest and currently most comprehensively excavated of the known localities along its frontiers. The wealth of material permits initial hypotheses to be formulated as to how the communities ideologically organised and expressed social organisation through their mortuary practices, and to do so from the perspective of the non-State, frontier pastoralists.

Re-assessing the growth and structure of the mortuary remains has led to a determination that the basic layout was conceived either at the outset or early on in its development (*contra* Gerharz 1994). This change in conceptualising how the valley was used permitted more detailed analyses to determine whether there are any patterns to the spread of wealth, burials and artefacts. While no valid clusters are detectable, the existence of a greater overall degree of wealth in the southwest sector combined with the spatial neighbourhood of likely influential individuals detected in the northeast sector is further suggestive of a degree of planning. These individuals should not automatically be assigned an elite status as the possibility remains to be discounted that they could have been from another social stratum such as smiths, which required people to be distanced from them in death as well as life. Interestingly, wealth does not appear to have been limited to any age or gender group, perhaps suggestive of a degree of wealth partly shared, at least in death, within families or lineages. However, there are more burials without accompanying goods in the northeast, perhaps suggestive of a sector of the population deriving legitimacy or social standing through association, and lending support to the idea that there is a form of hierarchical status being displayed in the mortuary domain.

There is no evidence for competing lineages demarcating different portions of the cemetery, as this would be reflected in the composition of the burial assemblages resulting in distinct spatial clusters. The ideology governing the social actions of the mortuary rites appear to have been fairly consistent in how the dead were represented through the accompanying burial items (or the lack thereof), as well which sector of the cemetery they were buried in. Any non-permanent markers and/or sandy burial mounds placed over the shallow burials would have eventually disintegrated, blending the burial into the natural landscape, and essentially transforming the individual into an eternal entity embedded in the sacred landscape through the actions and social decisions taken by the living.

The continued application of mortuary theory to the data obtained from statistical analysis will lead to further insights on the social organisation, reflected through issues of dominance, power, influence and social inversions enacted through performance rituals, as ideologically reflected in the burial assemblages. A more detailed study of these relations at Jebel Moya, a prominent massif in the otherwise fairly flat southern Gezira Plain, is underway to understand the social traits and values placed upon the material items by the groups’ social and behavioural processes. This and forthcoming studies will add to a previous call by Krzysztof Grzymski (2004, p. 24) to identify “ancient cultural constructs and meanings” in conceptual landscapes through detailed examination of how social practices were represented in the mortuary domain at Jebel Moya. Finally, further examination of the differences between the Jebel Moya and Meroitic cemeteries, from those of Sennar to those of Gabati and elsewhere in the Shendi Reach (Babiker 1985; Edwards 1998a, 1999; Suková and Cílek 2012), will help to shed light on not just the relationships between societies in the southwest frontier zone, but also on the social organisation as reflected in the respective mortuary assemblages.
